# Horizontal inequity in public health care service utilization for non-communicable diseases in urban Vietnam

**DOI:** 10.3402/gha.v7.24919

**Published:** 2014-08-04

**Authors:** Vu Duy Kien, Hoang Van Minh, Kim Bao Giang, Lars Weinehall, Nawi Ng

**Affiliations:** 1 Center for Health System Research, Hanoi Medical University, Hanoi, Vietnam; 2 Department of Public Health and Clinical Medicine, Unit of Epidemiology and Global Health, Umeå University, Umeå, Sweden; 3 Institute for Preventive Medicine and Public Health, Hanoi Medical University, Hanoi, Vietnam

**Keywords:** healthcare utilization, horizontal equity, non-communicable diseases, decomposition, urban Vietnam

## Abstract

**Background:**

A health system that provides equitable health care is a principal goal in many countries. Measuring horizontal inequity (HI) in health care utilization is important to develop appropriate and equitable public policies, especially policies related to non-communicable diseases (NCDs).

**Design:**

A cross-sectional survey of 1,211 randomly selected households in slum and non-slum areas was carried out in four urban districts of Hanoi city in 2013. This study utilized data from 3,736 individuals aged 15 years and older. Respondents were asked about health care use during the previous 12 months; information included sex, age, and self-reported NCDs. We assessed the extent of inequity in utilization of public health care services. Concentration indexes for health care utilization and health care needs were constructed via probit regression of individual utilization of public health care services, controlling for age, sex, and NCDs. In addition, concentration indexes were decomposed to identify factors contributing to inequalities in health care utilization.

**Results:**

The proportion of healthcare utilization in the slum and non-slum areas was 21.4 and 26.9%, respectively. HI in health care utilization in favor of the rich was observed in the slum areas, whereas horizontal equity was achieved among the non-slum areas. In the slum areas, we identified some key factors that affect the utilization of public health care services.

**Conclusion:**

Our results suggest that to achieve horizontal equity in utilization of public health care services, policy should target preventive interventions for NCDs, focusing more on the poor in slum areas.

A health system that provides equitable health care is a principal goal in many countries in the world. Horizontal equity in healthcare utilization is defined as ‘equal treatment for equal medical need, irrespective of other characteristics such as income, race, place of residence, etc.’ (1–3). Although several achievements in health care have been observed, the distribution of health care utilization is still inequitable (4, 5). Studies show that people’s health needs are not addressed equitably in many developed and developing countries (5–7). The evidence also suggests that after controlling for needs, health care distribution that benefits the rich greatly exceeds distribution that benefits the poor (6, 8, 9). Appropriate methods of measuring horizontal inequity (HI) in health care utilization play a key role in contributing to the development of appropriate public policies for health care systems (5, 7).

Since Vietnam’s economic reform in 1986, the country has achieved significant results in economic development. In addition, the health of the country’s population has improved substantially. Average life expectancy at birth increased from 67.8 years in 2000 to 73 years in 2012 (10, 11). The infant mortality rate decreased from 44.4 per 1,000 live births in 1990 to 15.4 per 1,000 live births in 2012 (11). However, Vietnam is suffering a double burden of diseases wherein communicable diseases still exist while non-communicable diseases (NCDs) are increasing (12). Along with the rapid economic growth, Vietnam has undergone a dramatic period of urbanization. The urban population increased from 24% in 1999 to 30% in 2009 (13).

The health care system in Vietnam is a mixed public–private provider system with the public sector dominating. The quality of health service delivery remains inadequate and poor for the population (12, 14, 15). Universal health coverage (UHC) aims to ensure that all people have access to health care services at affordable cost. In 2008, Vietnam approved a law on Social Health Insurance (SHI) to create a national SHI program, and in 2013 it developed a project to implement a roadmap toward UHC in 2012–2015 and a vision of UHC in 2020 (11). The Vietnam Living Standard Surveys have reported a gap between the rich and the poor in terms of health care utilization in different cross-sectional periods (16–19). Another study also reports that health care utilization in Vietnam changes in several years (15). There is however little information about horizontal inequality in health care utilization in urban areas, especially in slum areas. Thus, the aim of this study is to identify HI in health care utilization in urban slum area in Vietnam. In addition, we identify relevant health factors, including self-reported NCDs, associated with inequity in health care utilization.

## Methods

### Study setting

The study was carried out in Hanoi, the capital city of Vietnam, which has 29 districts of which 10 are urban and 19 are rural. Hanoi’s population is estimated to be 6.5 million, of which 2.6 million (41%) live in urban districts (20). In this study, we purposely selected four urban districts, namely Ba Dinh, Dong Da, Hai Ba Trung, and Hoan Kiem, as they included both slum and non-slum areas. We adapted a definition of the United Nations that slum areas are ‘those groups of households whose people live in temporary houses, insecure locations, and narrow spaces, or near a polluted environment’ (21).

### Study design and data collection

We conducted a population-based cross-sectional study in February–March 2013. Face-to-face interviews using structured questionnaires were conducted with the heads of households. The interviews were carried out by trained medical students of Hanoi Medical University and were supervised by senior staff of the Center for Health System Research, Hanoi Medical University.

### Sample size and sampling

The sample size was determined using a level of significance of 0.05 with a relative precision of 0.4, while an anticipated 10% of households had at least one member with a NCD (we estimate this prevalence in a pilot study of 60 households) (22). We control for a 30% non-response rate and design effects of 2 because of the use of cluster sampling. Thus, the targeted sample size is at least 600 households in either the non-slum or the slum areas. A mapping study was conducted to determine the list of 84 slum areas in the four urban districts. We randomly selected 30 slum areas from a list of 84 slum areas and 30 non-slum areas in the same commune by matching each area. In each area, we targeted a selection of approximately 20 households. A household is defined as comprising one person or a group of people who share accommodation and meals for at least 6 months during the past 12 months. The first household was randomly selected at the center of the slum or non-slum area, and the next household was the one adjacent to the previous. In each area, the data collections were concluded when the total number of interviewed households reached 20. In each selected households, all individuals 15 year old or over were invited to the study.

### Variables

To measure healthcare utilization, we asked about visits to public health facilities in the past 12 months prior to the interviews. Some people reported using private health care services; however, in this study, we focus only on those who reported accessing public health care services, which dominate the health care provision in Vietnam. In addition, public health care facilities participate fully in the national SHI scheme. Public health facilities include commune health centers, district hospitals, provincial hospitals, and national hospitals. In this study, we focus on the four main diseases that account for 80% of the total mortality from NCDs: cardiovascular diseases (heart disease or stroke), diabetes, chronic respiratory diseases (chronic obstructive pulmonary disease and asthma), and cancer (malignant tumors) (23).

### Measurement of wealth index

Since data for household income are not reliable, especially in developing countries, the wealth index, which is a composite index composed of key asset ownership variables were used as a proxy indicator for socioeconomic status (24, 25). We collected information on living conditions, including construction materials used in dwellings (e.g. materials for roofs, walls, and floors), access to utilities and infrastructure (e.g. sanitation facilities and sources of water), and ownership of selected durable assets (e.g. TVs, radios, computers, Internet access, telephones, mobile phones, VCD/DVD players, refrigerators, washing machines, water heaters, motorbikes, and cars). We applied principal components analysis (PCA) to construct a wealth index for our study population, separately for the slum and non-slum areas (26). The variables with multiple categories, such as material used in dwelling construction, were broken down into a set of dummy variables. Simple descriptive statistical analysis was performed to check the relevant variables; we used the rule of thumb that any variable with a prevalence below 5% or higher than 95% should be excluded from the analysis.

### Indirect health care need standardization

An indirect method was used to compare the differences between actual need and need standardized distributions for the probability of public health care utilization during a year. Non-linear estimation was used to account for the large proportion of observations with no utilization of public health care services (27). A probit model with control variables was used to estimate the difference between need predicted use and actual use. In addition, we used both ordinary least squares (OLS) and probit models to estimate need standardized health care use with and without controls.

The method proposed by O’Donnell et al. was applied to standardize indirect health care need (3, 27). The linear model of the relationship between, on one hand, public health care utilization and, on the other hand, need and control variables can be estimated by the following equation.

1 $$y_{i}=\alpha +\sum_{j}\beta_{j}x_{ji}+\sum_{k}\gamma_{k}z_{ki}+\varepsilon_{i}$$

where *y*_*i*_ is a public health care utilization variable; *i* denotes the individual; and *α*, *β*, and *γ* are parameter vectors ɛ is the error. *x*_*j*_ are confounding variables that we want to standardize, and *z*_*k*_ are non-confounding variables that we do not want to standardize but instead control in order to estimate partial correlations with the confounding variables. We used the OLS model to estimate the predicted value for public health care utilization by the following equation.

2 $${\hat{y}}_{i}^{x}=\hat{\alpha }+\sum_{j}{\hat{\beta }}_{j}x_{ji}+\sum_{k}{\hat{\gamma }}_{k}z_{ki}$$

where ${\hat{y}}_{i}^{x}$ is the predicted value of public health care utilization, and $\hat{\alpha }$, ${\hat{\beta }}_{j}$, and ${\hat{\gamma }}_{k}$ are OLS parameters. Finally, estimates of indirectly standardized public health care utilization ${\hat{y}}_{i}^{IS}$ are given by the difference between actual public health care utilization (*y*_*i*_) and predicted public health care utilization ${\hat{y}}_{i}^{x}$, plus the overall sample mean $\bar{y}$:

3 $${\hat{y}}_{i}^{IS}=y_{i}-{\hat{y}}_{i}^{x}+\bar{y}$$

The non-linear model of the relationship between public health care utilization, *y*, which is binary, and need (*x*) and control (*z*) variables in terms of a general functional form, *G*, is presented as follows:

4 $$y_{i}=G\left(\alpha +\sum_{j}\beta_{j}x_{ji}+\sum_{k}\gamma_{k}z_{ji}\right)+\varepsilon_{i}$$

where *G* takes particular forms for the probit model. Then the standardized need was estimated using the following equation (3).

5 $${\hat{y}}_{i}^{IS}=y_{i}-G\left(\hat{\alpha }+\sum_{j}{\hat{\beta }}_{j}x_{ji}+\sum_{k}{\hat{\gamma }}_{k}{\bar{z}}_{k}\right)+\frac{1}{n}\times \sum_{i=1}^{n}G\left(\hat{\alpha }+\sum_{j}{\hat{\beta }}_{j}x_{ji}+\sum_{k}{\hat{\gamma }}_{k}{\bar{z}}_{k}\right)$$

where *n* is the sample size and *z* variables are set to their means ${\bar{z}}_{k}$ to obtain the predictions. As noted above, ${\hat{y}}_{i}^{IS}$ is the standardized public health care utilization.

### Marginal effects

Since the outcome variable, self-reported NCDs, was binary with the range of (0,1), we applied the probit model to extract marginal effects of each determinant on observed probabilities of the outcome variable. The marginal effects provide evidence of associations between determinants and the outcome variable (self-reported NCDs). The marginal effects with positive signs indicate that they have positive association with the probability of self-reported NCDs, while those with negative signs indicate negative associations. A large absolute value of a marginal effect presents a strong association.

### Health inequality analysis

According to egalitarian principles, health care should be distributed based on a need principle rather than people’s willingness and ability to pay. It is suggested that if health care is allocated appropriately, health equity will be promoted. However, need is a very difficult concept to define and measure (1, 3). In this study, we used demographic characteristic variables (age–sex dummy variables) and NCDs as a proxy measure for need (Table 1). In order to measure inequity, inequality in public health care utilization was standardized for differences in need. After standardizing this inequality, any residual inequality in utilization by socioeconomic status (based on the wealth index) is interpreted as HI, which would be pro-rich if its value is positive and pro-poor if its value is negative.

**Table 1:** Variables included in this study

Categories	Variables
Healthcare utilization	The probability of any public healthcare utilization
Healthcare need	Sex–Age, self-reported of non-communicable diseases
Control variables	Wealth index, education, occupation, health insurance

To evaluate HI in our model, we applied the same method used to estimate the concentration index (CI) of actual health care use and health care need (1, 3). The value of HI is measured as the difference between the CI of health care use and that of health care need. The CI is directly related to the concentration curve, which plots the cumulative percentage of the health care utilization variable (on the y-axis) and the cumulative percentage of socioeconomic variables ranked from poorest to richest (on the x-axis). The CI is calculated as twice the area between the concentration curve and the line of equality (the 45° line). The CI ranges between −1 and +1. The CI takes a value of 0 if health care utilization distribution is completely equal. It is negative when the concentration curve lies above the line of equality, which indicates greater concentration of the health care utilization variable among the poor. Meanwhile, it takes a positive value if the concentration curve lies below the line of equality, which indicates greater concentration of the health care utilization variable among the rich. The CI of health care use (C_M_) was calculated by the following equation.

6 $$C=\frac{2}{\mu }Cov(h,r)$$

where *µ* is the mean of the health outcome, *h* is health care utilization of the individual, and *r* is rank of the individual by wealth distribution (1). Similarly, if we replace *h* with the health care need of the individual, we can calculate the CI of health care needs (C_N_). The HI was calculated by subtracting *C*_*N*_ from *C*_*M*_. A positive value of HI indicates a distribution of health care utilization in favor of the rich, and vice versa.

7 $$HI_{WV}=C_{M}-C_{N}$$

where HI_WV_ is a horizontal index using the indirect standardization approach. The CI of public health care utilization can be decomposed into the contribution of determinants based on the linear function in Equation 1. The CI for *y* can be written as

8 $$C=\sum_{j}(\beta_{j}{\bar{x}}_{j}/\mu )C_{j}+\sum_{k}(\gamma_{k}{\bar{z}}_{k}/\mu )C_{k}+GC_{S}/\mu$$

If a non-linear model is used, then decomposition is possible only if some linear approximation to the non-linear model is made. One possibility is to use estimates of the partial effects evaluated at the means. That is, a linear approximation to Equation 4 is given by

9 $$y_{i}=\alpha^{m}+\sum_{j}\beta_{j}^{m}x_{ji}+\sum_{k}\gamma_{k}^{m}z_{ki}+\varepsilon_{i}$$

where $\beta_{j}^{m}$ and $\gamma_{k}^{m}$ are the partial effects of each variable treated as a fixed parameter and evaluated at the sample means, and *ɛ*_*i*_ is the error, which includes approximation errors. Then, the CI for *y* for this non-linear approach can be written as

10 $$C=\sum_{j}(\beta_{j}^{m}{\bar{x}}_{j}/\mu )C_{j}+\sum_{k}(\gamma_{k}^{m}{\bar{z}}_{k}/\mu )C_{k}+GC_{S}/\mu$$

where *µ* is the mean of y; ${\bar{x}}_{j}$ is the mean of *x*_*j*_; and ${\bar{z}}_{k}$ is the mean of *z*_*k*_, GC_ɛ_ is the generalized concentration index for the error term (ɛ) (3).

Data were analyzed using Stata statistical software version 12.1. The level of statistical significance was set to 0.05. Missing data were excluded from the data analysis.

### Ethical considerations

The protocol for this study has been approved by the Scientific and Ethical Committee in Biomedical Research, Hanoi Medical University. All human subjects in the study were asked for their consent, and they had provided their consent before data collection, and all had complete rights to withdraw from the study at any time without threats or disadvantages.

## Results

A total of 1,211 households were included in the study (600 and 611 households in the non-slum and slum areas, respectively). The non-response rate, about 3–5%, is quite similar in both slum and non-slum areas. Our inclusion criteria for participants are those who are 15 years old or older, and this generates a total of 3,815 people in our sample.

### Poor–rich distribution of public health care utilization and their determinants

Table 2 presents the proportion and CI for public health care utilization. The self-reported public health care utilization variables show that people in both slum and non-slum areas used mainly national hospitals. In general, 21.3% of people in slum areas reported visiting any public health care facility during the past 12 months while 26.7% of those in non-slum areas reported the same. The crude concentration indexes for all levels and any public health care utilization for the non-slum areas were negative, indicating that health care utilization was concentrated among the poor in the non-slum areas. In the slum areas, the crude concentration indexes for visiting district (C=−0.063) and provincial hospitals (C=−0.112) were negative, indicating that at district and provincial hospitals, health care utilization was concentrated among the poor. However, the crude concentration indexes for communal health centers (C=0.044) and national hospitals (C=0.114) were positive, indicating that at these levels, health care utilization was concentrated among the rich in the slum areas.

**Table 2:** Proportion and concentration indexes of public healthcare utilization during the past 12 months and the distributions of their determinants for slum and non-slum areas in Hanoi

	Slum areas		Non-slum areas	
	
Variables	Proportion (in decimal)	Concentration index	Proportion (in decimal)	Concentration index
Public healthcare utilization				
Communal health center	0.020	0.044	0.017	−0.157
District hospitals	0.035	−0.063	0.038	−0.020
Provincial hospitals	0.053	−0.112	0.073	−0.049
National hospitals	0.118	0.114	0.148	−0.101
Any public healthcare utilization	0.213	0.034	0.267	−0.074
Age–sex				
Males aged 15–29	0.137	0.016	0.090	0.121
Males aged 30–44	0.124	0.053	0.133	0.079
Males aged 45–59	0.109	−0.016	0.110	−0.014
Males aged 60+	0.094	0.069	0.137	−0.115
Females aged 15–29	0.135	0.055	0.125	0.068
Females aged 30–44	0.148	−0.041	0.135	0.061
Females aged 45–59	0.137	−0.083	0.118	−0.016
Females aged 60+	0.117	−0.029	0.152	−0.126
Wealth index				
Wealth quintile: 1 – lowest 20%	0.167	−0.833	0.161	−0.839
Wealth quintile: 2 – lower 20%	0.180	−0.486	0.214	−0.465
Wealth quintile: 3 – middle 20%	0.209	−0.098	0.197	−0.054
Wealth quintile: 4 – higher 20%	0.212	0.323	0.230	0.373
Wealth quintile: 5 – highest 20%	0.233	0.767	0.199	0.801
Education				
Education: primary school or less	0.187	−0.381	0.060	−0.270
Education: secondary school	0.281	−0.151	0.181	−0.119
Education: high school	0.282	0.099	0.271	0.008
Education: college/university or higher	0.250	0.345	0.487	0.073
Work status				
Work status: professionals, technicians, or social services	0.139	0.300	0.238	0.139
Work status: worker, farmer, or crafts worker	0.178	−0.029	0.124	−0.061
Work status: self-employed	0.289	−0.149	0.187	−0.039
Not in workforce: unemployed	0.053	−0.315	0.025	−0.253
Not in workforce: retired	0.204	0.073	0.310	−0.083
Not in workforce: other, such as not decided to work or student/pupil	0.138	0.060	0.116	0.120
Non-communicable diseases	0.079	−0.077	0.116	−0.169
Health insurance	0.658	0.109	0.826	0.013

The proportions for categories of determinants in Table 2 showed distributions of respondents across these categories. The crude concentration indexes showed the poor–rich distribution of the determinants. In the non-slum areas, people aged 60 years or older were strongly concentrated among the poor (C=−0.115 for males, C=−0.126 for females). In contrast, in the slum areas, people aged 60 years or older were concentrated among the poor for only females (C=−0.029), but among the rich for males (C=0.069). For those aged 45–59 years, both sexes were concentrated among the poor in both the non-slum and slum areas. The socioeconomic status inequality gradient can be seen clearly; people in lower wealth quintiles were concentrated more among the poor, while people in higher wealth quintiles were concentrated more among the rich. In addition, the socioeconomic gradient in education was clear. Those who graduated from secondary school or less were poorer, while those who graduated from high school or more were richer. Those who were manual workers, self-employed, or unemployed were concentrated among the poor. In the slum areas, about 8% of respondents who reported having NCDs were concentrated among the poor (C=−0.077). In non-slum areas, about 12% of respondents who reported having NCDs were concentrated among the poor (C=−0.169) in the non-slum areas. About 83% of people are covered by health insurance in the non-slum areas, but only 66% of people are covered by health insurance in the slum areas. Those with health insurance were concentrated among the rich in both non-slum (C=0.109) and slum areas (C=0.013).

### Marginal effects of determinants

Table 3 shows the marginal effects of each determinant on each public health care utilization variable. Increasing age in both men and women was significantly associated with increased probabilities of reporting public health care utilization, and the effects were consistently largest for respondents aged 60 years or older in both slum and non-slum areas. There was no association between wealth quintiles and the probability of reporting public health care utilization. However, NCDs and the presence of health insurance had strong positive associations with the probability of public health care utilization.

**Table 3:** Probability of determinants on any public healthcare utilization during the past 12 months in slum and non-slum areas in Hanoi (marginal effect using the probit model)

Variables	Slum areas	Non-slum areas
Age–sex		
Males aged 15–29	ref.	ref.
Males aged 30–44	−0.022	0.034
Males aged 45–59	0.101*	0.190**
Males aged 60+	0.272***	0.363***
Females aged 15–29	−0.066	−0.023
Females aged 30–44	0.032	0.065
Females aged 45–59	0.234***	0.362***
Females aged 60+	0.212***	0.464***
Wealth index		
Wealth quintile: 1 – lowest 20%	ref.	ref.
Wealth quintile: 2 – lower 20%	0.039	0.168
Wealth quintile: 3 – middle 20%	−0.008	−0.003
Wealth quintile: 4 – higher 20%	0.048	0.016
Wealth quintile: 5 – highest 20%	0.031	−0.003
Education		
Education: primary school or less	ref.	ref.
Education: secondary school	−0.005	−0.082*
Education: high school	0.009	0.019
Education: college/university or higher	−0.021	−0.008
Work status		
Work status: professionals, technicians, or social services	ref.	ref.
Work status: worker, farmer, or craft worker	0.030	0.028
Work status: self-employed	0.050	0.033
Not in workforce: unemployed	0.090	0.066
Not in workforce: retired	0.093*	0.052
Not in workforce: other, such as not decided to work or student/pupil	−0.008	−0.035
Non-communicable diseases		
No	ref.	ref.
Yes	0.268***	0.229***
Health insurance coverage		
No	ref.	ref.
Yes	0.113***	0.115***

Dependent variable for the probit model is a dichotomous indicator of whether a person has had any health care during the past 12 months or not, and indicates significance level as follows:

*** p≤0.001,

** 0.001<p≤0.01,

* 0.01<p≤0.05.

### Horizontal equity

In the slum areas, as Table 4 shows, the results of our estimation clearly suggested that the actual distribution observed was pro-rich (a higher mean among the wealthiest) and the need expected distribution was pro-poor in the slum areas (a higher mean among the poorest). This was because ‘need’, as proxied by demographics and NCDs, was concentrated more among the lower socioeconomic status groups in the slum areas. After using need standardization, the mean of the wealthiest 20% using public health care services was 0.217, which was 26% higher than the mean of the poorest 20% (0.172). On the contrary, for the non-slum areas, as Table 5 shows, we found that actual and need predicted public health care utilization were concentrated more among the poor. This means that in the non-slum areas, the poor were more likely to have the opportunity to use public health care services. Thus, we do not take our analysis further for the non-slum areas.

**Table 4:** Distributions of actual, need predicted, and need standardized healthcare utilization in slum areas in Hanoi

Probability of any public health care utilization							

				Need standardized			
		
	Probit with controls			With controls		Without controls	
	
Quintiles	Actual	Need predicted	Different=predicted−actual	Probit	OLS	Probit	OLS
Wealth quintile: 1 – lowest 20%	0.174	0.201	−0.027	0.172	0.172	0.176	0.176
Wealth quintile: 2 – lower 20%	0.223	0.213	0.011	0.210	0.210	0.207	0.207
Wealth quintile: 3 – middle 20%	0.203	0.196	0.009	0.207	0.206	0.205	0.205
Wealth quintile: 4 – higher 20%	0.255	0.196	0.060	0.259	0.259	0.259	0.259
Wealth quintile: 5 – highest 20%	0.208	0.190	0.018	0.217	0.217	0.217	0.217
Mean	0.213	0.199	0.014	0.213	0.213	0.213	0.213
Concentration index/*HI*_*WV*_	0.034	−0.019		0.052	0.050	0.050	0.050
Standard error	0.028	0.010		0.025	0.025	0.025	0.025
t-ratio	1.214	−1.889		2.033	1.998	1.995	2.011

OLS=Ordinary Least Square model; probit=probit model; HI_WV_=horizontal inequity index using the indirect standardization approach.

**Table 5:** Distributions of actual, need predicted, and need standardized healthcare utilization in non-slum areas in Hanoi

Probability of any public health care utilization							

				Need standardized			
				
	Probit with controls			With controls		Without controls	
	
Quintiles	Actual	Need predicted	Different=predicted−actual	Probit	OLS	Probit	OLS
Wealth quintile: 1 – lowest 20%	0.335	0.313	0.022	0.280	0.281	0.273	0.273
Wealth quintile: 2 – lower 20%	0.271	0.250	0.021	0.278	0.278	0.278	0.279
Wealth quintile: 3 – middle 20%	0.222	0.246	−0.024	0.234	0.234	0.236	0.236
Wealth quintile: 4 – higher 20%	0.262	0.239	0.022	0.280	0.280	0.281	0.282
Wealth quintile: 5 – highest 20%	0.243	0.237	0.006	0.263	0.262	0.265	0.265
Mean	0.267	0.257	0.010	0.267	0.267	0.267	0.267
Concentration index/*HI*_*WV*_	−0.075	−0.066		−0.011	−0.013	−0.004	−0.004
Standard error	0.022	0.010		0.019	0.019	0.019	0.019
t-ratio	−3.364	−6.456		−0.580	−0.667	−0.222	−0.234

OLS=Ordinary Least Squares; HI_WV_=horizontal inequity index using the indirect standardization approach.

For the slum areas, we extend our analysis to decompose the effects of need factors and non-need factors in the utilization of public health care services. Table 6 shows that the contribution of each need factor was negative, indicating that if utilization was determined by need factors alone, it would be pro-poor. After standardizing the percentage of contribution, the aggregate contribution of all need factors decreased the unstandardized CI by about 61%. When we examined the need factors in detail, the distribution of age–sex groups and NCDs pushed utilization in a pro-poor direction by about 40 and 21%, respectively. In addition, if needs were distributed equally, the direct effect of the wealth index and health insurance coverage on health care utilization would increase the unadjusted CI by approximately 37 and 54%, respectively, which pushed utilization in a pro-rich direction. At the same time, if needs were distributed equally, education and work status reduced the unadjusted CI by approximately 8 and 31%, respectively, thus pushed the unadjusted CI in a pro-poor direction.

**Table 6:** Decomposition of concentration index for public health care utilization in slum areas in Hanoi

Contributions to concentration index for any healthcare utilization						

	OLS			Probit partial effects		
	
	Absolute	Percentage	Standardized percentage	Absolute	Percentage	Standardized percentage
Need factors						
Age–sex groups	−0.010	−29.05	−34.02	−0.015	−43.47	−39.86
Non-communicable diseases	−0.008	−24.43	−28.61	−0.008	−22.78	−20.89
Subtotal	−0.018	−53.48	−62.63	−0.023	−66.25	−60.75
Non-need factors						
Wealth index	0.026	76.81	41.88	0.026	76.76	36.88
Health insurance coverage	0.036	106.59	58.12	0.038	113.17	54.38
Education	−0.003	−8.85	−10.36	−0.003	−8.55	−7.84
Work status	−0.007	−20.03	−23.46	−0.012	−34.25	−31.41
Subtotal	0.052	154.52	84.25	0.050	147.13	70.70
Residual	−0.001	−3.03	−3.55	0.006	18.18	8.74
Total	0.033			0.033		
Horizontal inequity index (HI_WV_)	0.052			0.056		

OLS=Ordinary Least Square model; probit=probit model; HI_WV_=horizontal inequity index using the indirect standardization approach.

## Discussion

The results of this study show evidence of inequities in public health care utilization that benefit better-off people in the slum areas in urban Hanoi in Vietnam. This is despite several policies of the government of Vietnam that support the poor and near-poor population and promote the implementation of health insurance for all people (14). In the slum areas, our analysis of the distribution of actual need, predicted need, standardized need, and the decomposition index all provide evidence of inequities in the use of medical care that benefit better-off people. In addition, our results indicate that the poor in the slum areas use less public health care services than expected based on their needs. This analysis of HIs in public health care utilization supplements our probit model analysis, which focuses on variables affecting public health care utilization, including sex–age, NCDs, and health insurance. However, the results show that public health care utilization benefits the poor in the non-slum areas, indicating that there is no evidence of an inequity problem in public health care utilization in the non-slum areas. Use of the equity analysis adopted by this study could provide important information for Vietnamese policy makers concerned with equity in public health care utilization, especially for the slum areas in Hanoi.

The probit model estimation shows some key factors that affect the utilization of public health care services. We find that the major predictors of service utilization in both slum and non-slum areas are sex–age, NCDs, and health insurance. This analysis provides evidence that older men and women are more likely to use health care services than younger people. In addition, self-reported NCDs are strongly related to increased public health care utilization. There is no difference by education (except the secondary school group in the non-slum areas) and work status (except the retired group in the slum areas). Although there is evidence suggesting that the rich use health care more than the poor (28, 29), this finding is not consistent across all wealth quintile groups.

We note some limitations of this study. First, the self-reported recall period of one year related to health care utilization in the questionnaires might suffer from the recall bias. Second, the cross-sectional nature of our study prevents interpretation of casual relationships. Therefore, a longitudinal study to monitor and measure health care utilization in terms of inequity in urban areas should be conducted to provide more robust evidence for policy development.

## Conclusion and recommendations

The findings of our study could help identifying targets for policies to improve equity in the use of public health care services in urban areas in Vietnam. We observed HI in health care utilization in favor of the rich in the slum areas, whereas horizontal equity was achieved in the non-slum areas. In particular, in the slum areas, decomposition of the CI demonstrated that self-reported NCDs contributed to push health care utilization in a pro-poor direction. Our results suggest that to achieve horizontal equity in the utilization of public health care services, policy should focus on preventive interventions for NCDs and on the poor population in slum areas. Moreover, even though SHI has been designed as a financial mechanism for reducing inequity in health care utilization; our results show that SHI contributes to pushing utilization in a pro-rich direction. Hence, appropriate policy should be considered to improve the health care access of people in slum areas by using SHI.

About half of the world’s population lived in urban areas by the end of 2008. Two thirds of the world’s population will live in urban areas in the next 30 years (30). Most of the global urban population will grow in the cities of developing countries, including the ASEAN countries (31). Rapid urbanization will create major challenges for the health care system, particularly when the burden of NCDs is increasing. Hence, setting up an appropriate system to monitor inequity in health care utilization, particularly on utilization related to NCDs, over time may help to understand the impact and implications of policies in the health care sector. These monitoring systems may help ASEAN countries keep track of their progress toward UHC.
